# Supplementary material for: DprE2 is a molecular target of the anti-tubercular nitroimidazole compounds pretomanid and delamanid
Source: Nat Commun. 2023 Jun 28;14:3828. doi: 10.1038/s41467-023-39300-z (PMC10307805; doi:10.1038/s41467-023-39300-z)
Supplement: Supplementary file 1 — Supplementary Information [file 41467_2023_39300_MOESM1_ESM.pdf]

Supplementary Information

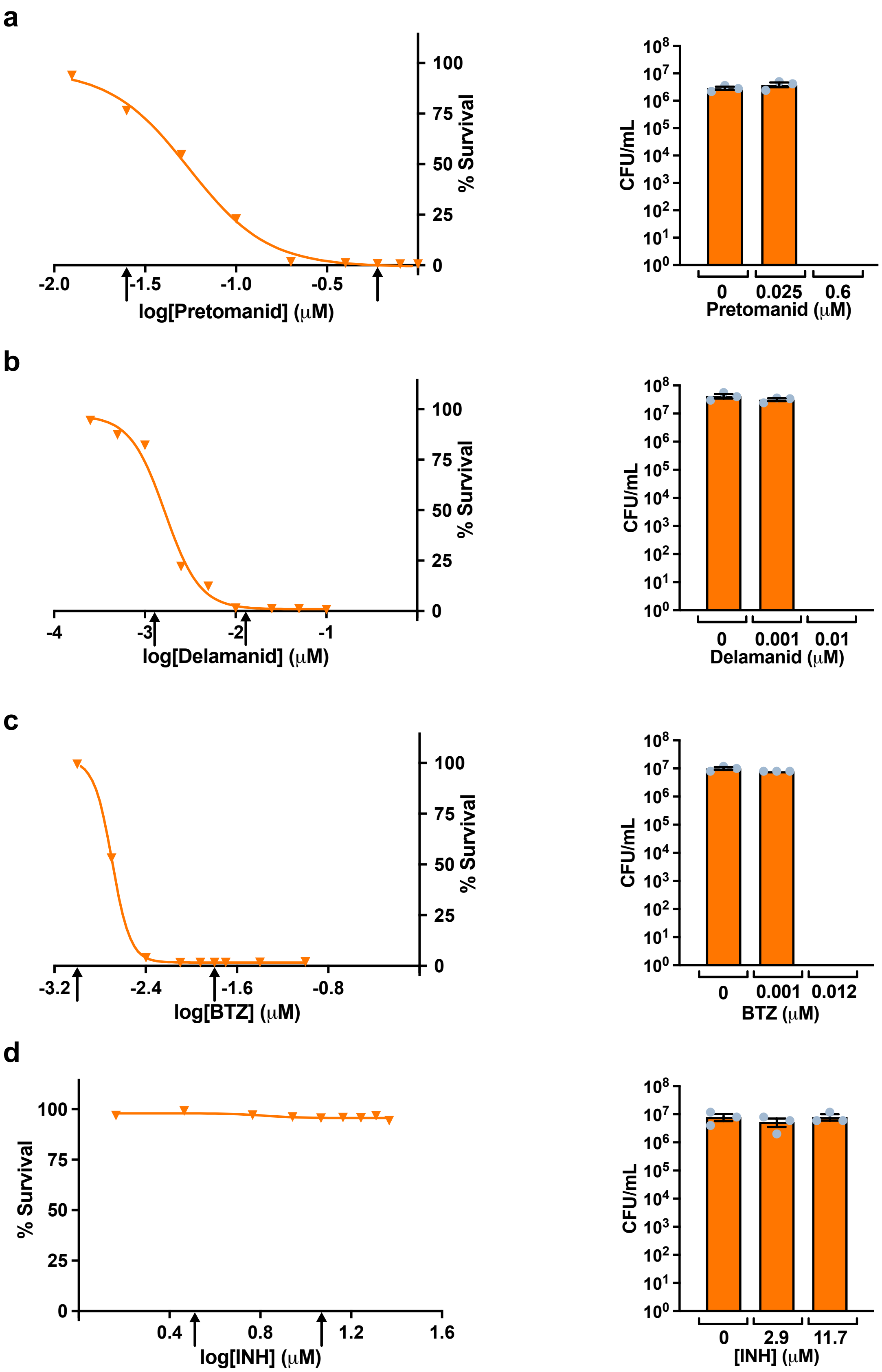

Supplementary Fig. 1: Over-expression of the NADH-dependent *Mtb* InhA does not confer resistance to pretomanid and delamanid. The MIC of **a** pretomanid, **b** delamanid, **c** BTZ and **d** INH were established using *M. bovis* BCG strain containing the constitutive expression vector pMV261-*inhA*. Cell viability was determined using the MABA. Arrows on the MABA graphs indicate the concentration of drug selected for CFU enumeration. Data were plotted and fitted using Prism GraphPad showing the percentage survival (MABA) or CFU/mL (individual data points shown as gray circles), based on the mean of triplicate data (*n* = 3) from independent experiments with bars representing the standard error. Concentrations used are equivalent to those in the main text. Source data are provided as a Source Data file.

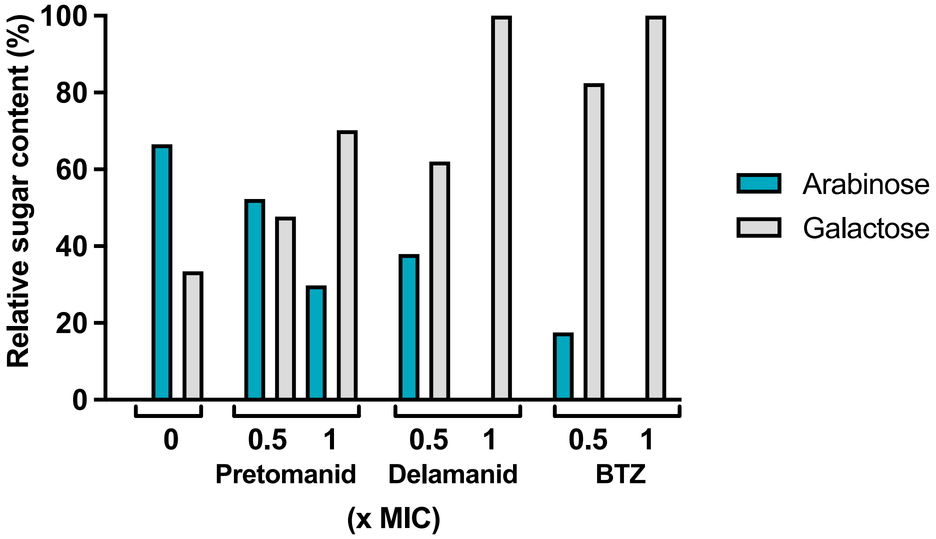

Supplementary Fig. 2: Bar graphs representing the effects of pretomanid, delamanid and BTZ treatment on the sugar components of arabinogalactan. *M. smegmatis* pVV16-*ddn* was treated with 0x, 0.5x, and 1x MIC pretomanid, delamanid or BTZ and labeled with [^{14}C]-D-glucose. The arabinogalactan was extracted, and the individual sugars were hydrolyzed prior to analysis by autoradiography TLC and densitometry. The bars (n = 1) represent the relative proportion of arabinose and galactose with respect to each other at the defined drug concentrations. There is a dose-dependent reduction in arabinose relative to galactose with treatment of pretomanid or delamanid, concomitant with the inhibition of DprE2 activity and DPA synthesis. This result is corroborated by the impact of BTZ on the cell wall D-arabinose, which is a known target of DprE1. Source data are provided as a Source Data file.

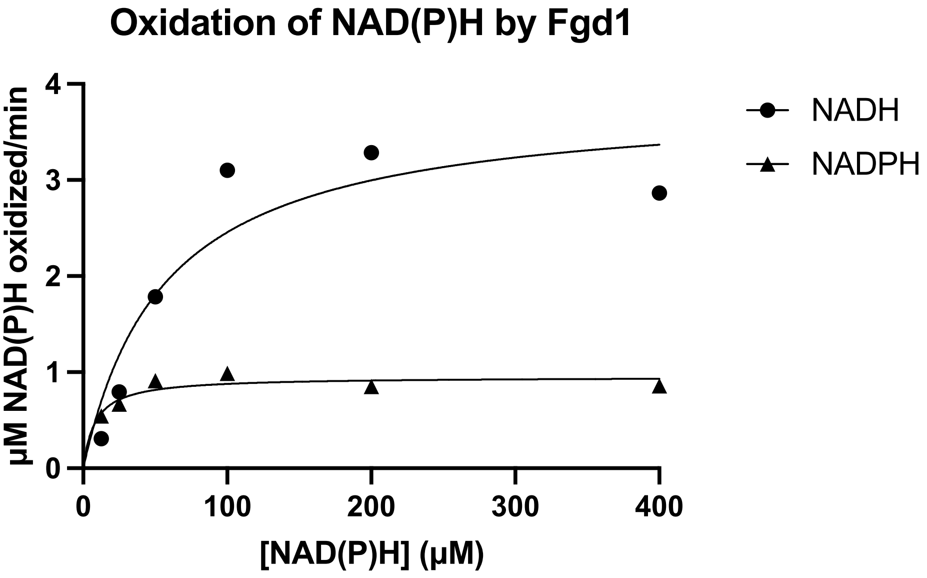

Supplementary Fig. 3. Oxidation of NAD(P)H by Fgd1. The components used to activate pretomanid were assayed for NAD(P)H oxidation with increasing concentrations of both cofactors in the presence and absence of Fgd1. Background oxidation was subtracted using the minus Fgd1 curves. Assays were performed in duplicate (n = 2) and mean initial rates (μM NAD(P)H oxidized/min), with the standard error, were plotted using Prism GraphPad. Source data are provided as a Source Data file.

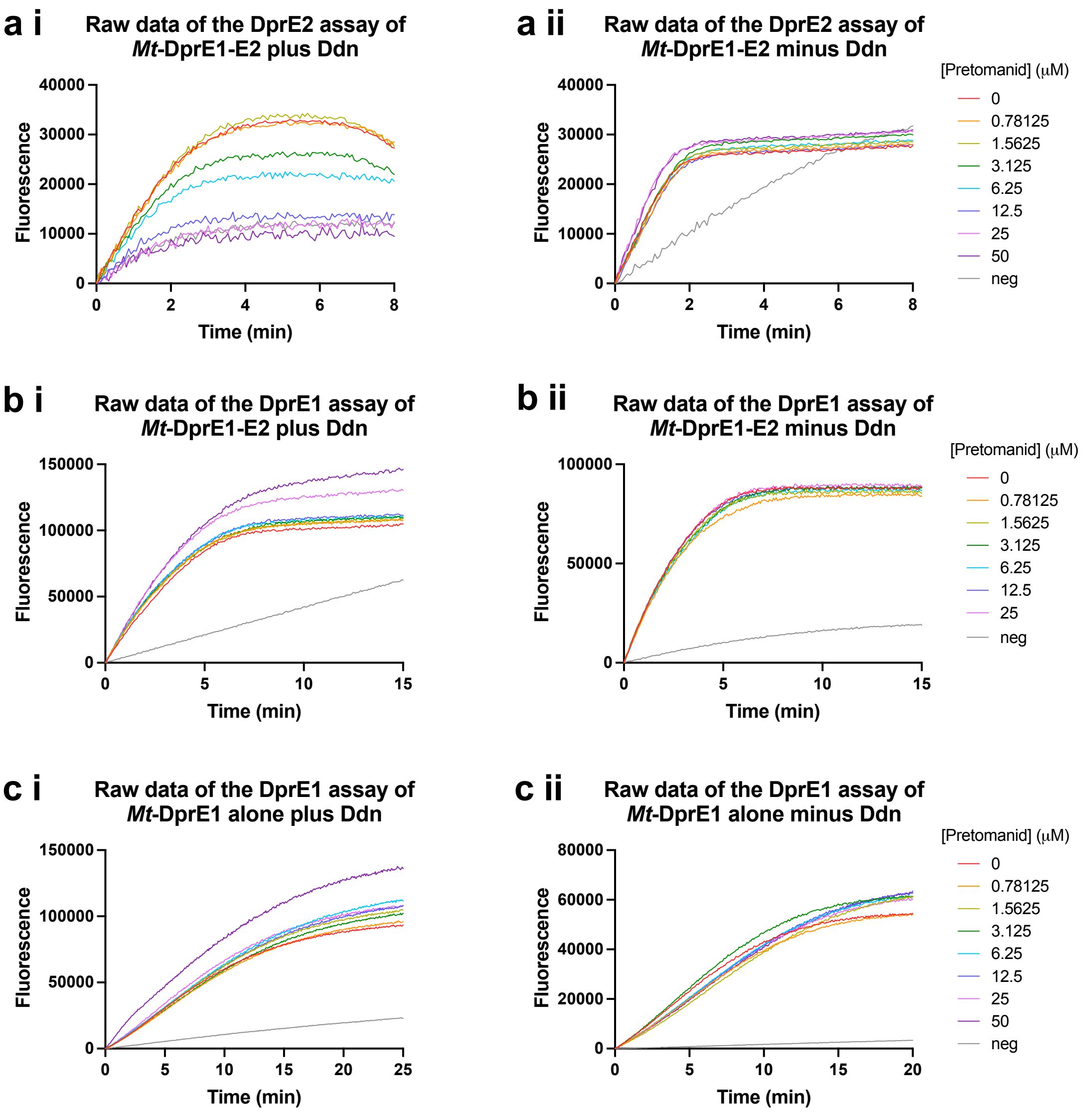

Supplementary Fig. 4. Raw data of pretomanid inhibition. a DprE2 assay of the *Mt*-DprE1-DprE2 complex, **b** DprE1 assay of the *Mt*-DprE1-DprE2 complex and **c** DprE1 assay of *Mt*-DprE1 alone, (i) plus and (ii) minus the pretomanid activating enzyme Ddn. Both assays use fluorescence intensity: the DprE2 assay tracks the oxidation of NADPH, while the DprE1 assay follows the reduction of resazurin. The negative control (neg; grey line) for each assay represents the same conditions as 0 pretomanid, omitting the GGPR (geranylgeranylphosphoryl-D-ribose) substrate. *Mt*-DprE1-E2, *Mt*-DprE1-DprE2 complex. Source data are provided as a Source Data file (Raw data from Fig. 4).

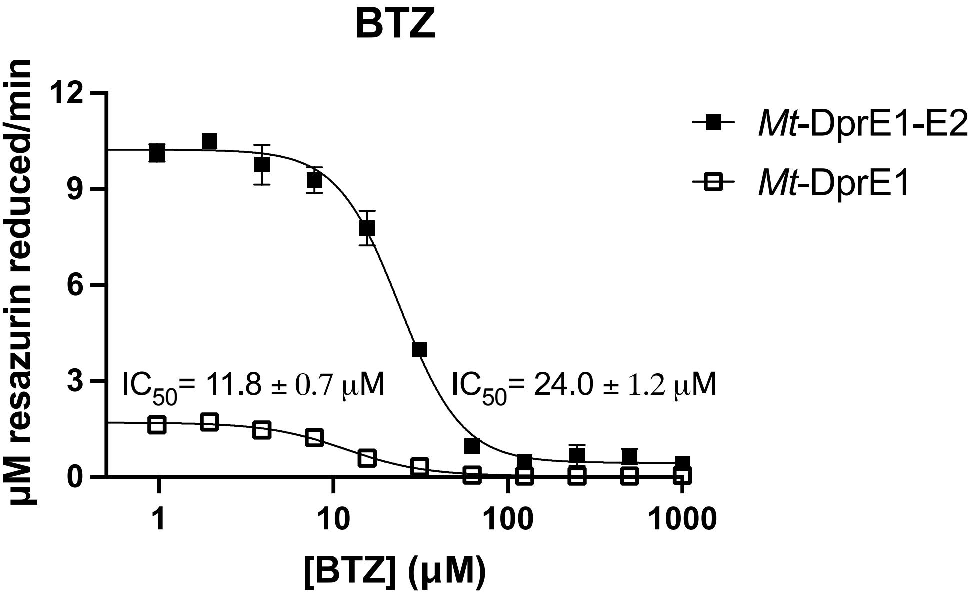

Supplementary Fig. 5. Inhibition of the DprE1 assay by BTZ. DprE1 assay monitoring BTZ inhibition of both the *Mt*-DprE1-DprE2 complex and *Mt*-DprE1 alone. Assays were performed in triplicate ($n = 3$) and graphs were plotted as [inhibitor] (log_{10} scale μM) vs. response (mean initial rate with SEM) and IC_{50} (± SEM) were calculated fitting a four-parameter dose response curve, using Prism GraphPad. *Mt*-DprE1-E2, *Mt*-DprE1-DprE2 complex. Source data are provided as a Source Data file.

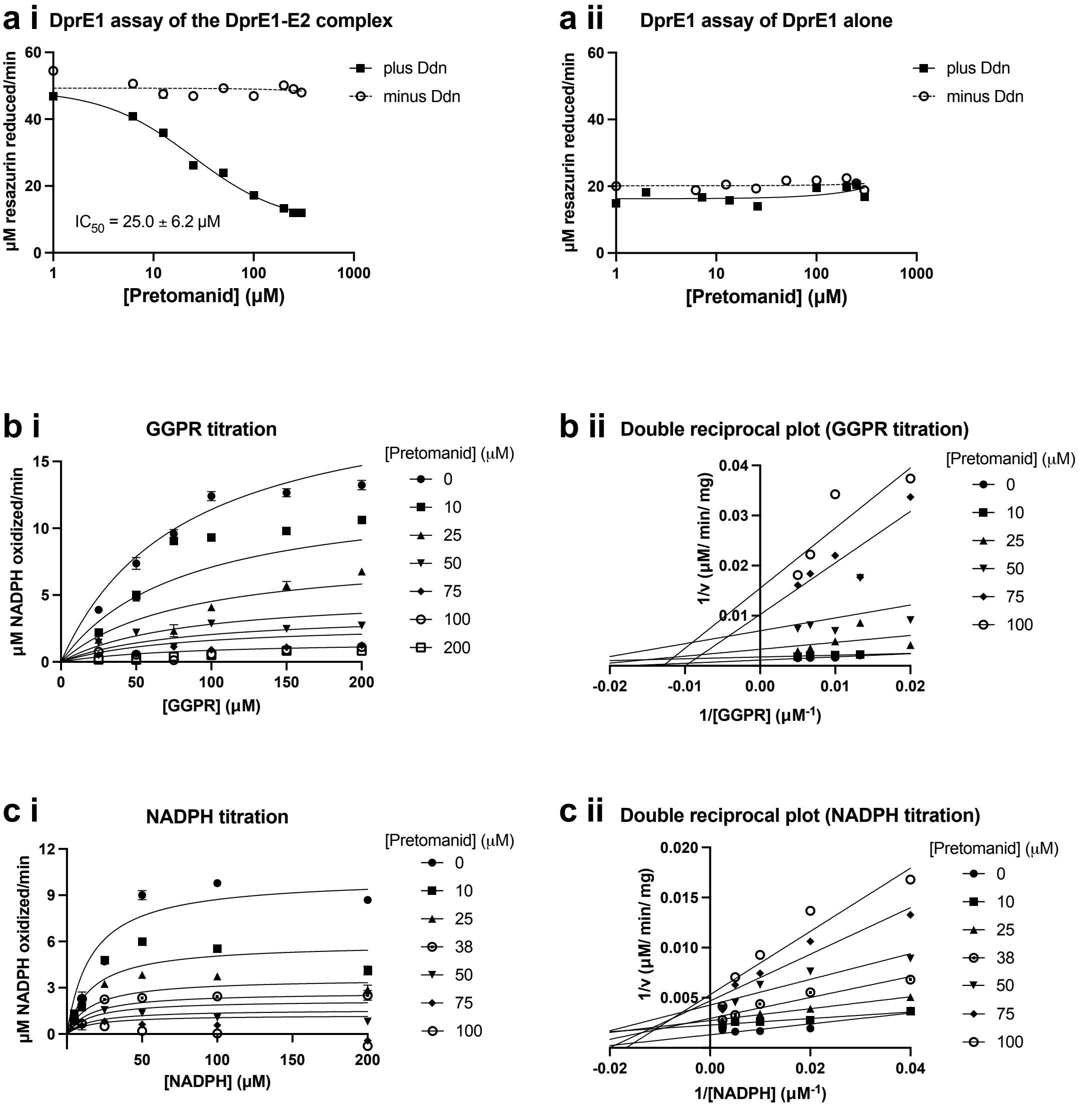

Supplementary Fig. 6. Enzyme kinetics for pretomanid inhibition during activation. *Mt*-DprE1/ *Mt*-DprE1-DprE2 were incubated for 3 hours at 30°C during pretomanid activation with all the activation components plus and minus Ddn. The NAD(H) required for pretomanid activation is speculated to be endogenous in the complex. **a** DprE1 assay monitoring DprE1 activity in (i) *Mt*-DprE1-DprE2 complex and (ii) *Mt*-DprE1 alone, with increasing concentrations of pretomanid. Assays were initiated with 200 μM GGPR. All assays were performed in triplicate (n = 3) and graphs were plotted [inhibitor] (log_{10} scale /μM) vs. response (mean initial rate with

SEM) and IC_{50} (± SEM) was calculated fitting a four-parameter dose response curve, using Prism GraphPad. K_i determination for **b** GGPR and **c** NADPH. Pretomanid inhibition was performed with varying concentrations of inhibitor and the two substrates. *Mt*-DprE1-DprE2 was incubated for 3 hours at 30°C with the activation components and increasing concentrations of pretomanid, with either 100 µM or a titration of NADPH. DprE2 activity was measured after the addition of 200 µM or a titration of GGPR. Assays were performed in triplicate ($n = 3$) and mean initial rates (µM NADPH oxidized/min), with the standard error, were plotted using Prism GraphPad, fitting the curve using the model for non-competitive inhibition. (i) The fitted curve and (ii) double reciprocal plot. Background NADPH oxidation was subtracted, except for the double reciprocal plot of the NADPH data. GGPR, geranylgeranylphosphoryl-D-ribose; *Mt*-DprE1-E2, *Mt*-DprE1-DprE2 complex. Source data are provided as a Source Data file.
