# Supplementary material for: DprE2 is a molecular target of the anti-tubercular nitroimidazole compounds pretomanid and delamanid
Source: Nat Commun. 2023 Jun 28;14:3828. doi: 10.1038/s41467-023-39300-z (PMC10307805; doi:10.1038/s41467-023-39300-z)
Supplement: Supplementary file 3 — Reporting Summary [file 41467_2023_39300_MOESM3_ESM.pdf]

nature portfolio

Corresponding author(s): G. S. Besra

Last updated by author(s): 18/05/23

Reporting Summary

Nature Portfolio wishes to improve the reproducibility of the work that we publish. This form provides structure for consistency and transparency in reporting. For further information on Nature Portfolio policies, see our Editorial Policies and the Editorial Policy Checklist.

Statistics

For all statistical analyses, confirm that the following items are present in the figure legend, table legend, main text, or Methods section.

n/a	Confirmed	
☐	☒	The exact sample size (n) for each experimental group/condition, given as a discrete number and unit of measurement
☐	☒	A statement on whether measurements were taken from distinct samples or whether the same sample was measured repeatedly
☒	☐	The statistical test(s) used AND whether they are one- or two-sided<br>*Only common tests should be described solely by name; describe more complex techniques in the Methods section.*
☐	☒	A description of all covariates tested
☐	☒	A description of any assumptions or corrections, such as tests of normality and adjustment for multiple comparisons
☐	☒	A full description of the statistical parameters including central tendency (e.g. means) or other basic estimates (e.g. regression coefficient) AND variation (e.g. standard deviation) or associated estimates of uncertainty (e.g. confidence intervals)
☒	☐	For null hypothesis testing, the test statistic (e.g. F, t, r) with confidence intervals, effect sizes, degrees of freedom and P value noted<br>*Give P values as exact values whenever suitable.*
☒	☐	For Bayesian analysis, information on the choice of priors and Markov chain Monte Carlo settings
☒	☐	For hierarchical and complex designs, identification of the appropriate level for tests and full reporting of outcomes
☒	☐	Estimates of effect sizes (e.g. Cohen's d, Pearson's r), indicating how they were calculated

Our web collection on statistics for biologists contains articles on many of the points above.

Software and code

Policy information about availability of computer code

Data collection	Microsoft Excel 16.66.1
Data analysis	Microsoft Excel 16.66.1<br>GraphPad Prism 9.5.1

For manuscripts utilizing custom algorithms or software that are central to the research but not yet described in published literature, software must be made available to editors and reviewers. We strongly encourage code deposition in a community repository (e.g. GitHub). See the Nature Portfolio guidelines for submitting code & software for further information.

Data

Policy information about availability of data

All manuscripts must include a data availability statement. This statement should provide the following information, where applicable:

- Accession codes, unique identifiers, or web links for publicly available datasets
- A description of any restrictions on data availability
- For clinical datasets or third party data, please ensure that the statement adheres to our policy

The data generated in this study (Fig. 2, fluorescence counts and CFUs; Fig. 3, uncropped TLC; Figs. 4 and 5, fluorescence counts; Supplementary Fig. 1, fluorescence counts and CFUs; Supplementary Fig. 2, densitometry values; Supplementary Figs. 3, 4, 5 and 6, fluorescence counts) are provided with this paper in the Source Data file.

Human research participants

Policy information about studies involving human research participants and Sex and Gender in Research.

Reporting on sex and gender	N/A
Population characteristics	N/A
Recruitment	N/A
Ethics oversight	N/A

Note that full information on the approval of the study protocol must also be provided in the manuscript.

Field-specific reporting

Please select the one below that is the best fit for your research. If you are not sure, read the appropriate sections before making your selection.

- [x] Life sciences
- [ ] Behavioural & social sciences
- [ ] Ecological, evolutionary & environmental sciences

For a reference copy of the document with all sections, see nature.com/documents/nr-reporting-summary-flat.pdf

Life sciences study design

All studies must disclose on these points even when the disclosure is negative.

Sample size	No sample size calculation was performed. Kinetic assays (with the exception of Supplementary Fig.3) and whole cell over-expression data were performed with 3 technical repeats and with multiple biological repeats. The 14C assay was performed with 3 biological repeats. The reproducibility of the biological repeats determined that these sample sizes were sufficient. The 14C whole cell labeling (Supplementary Fig. 2) was performed in singlet and Fgd1 kinetic assay (Supplementary Fig. 3) was performed in duplicate due to substrate availability and time limitation.
Data exclusions	Data were excluded on observing anomalous results (deviating from technical and biological repeats) and were omitted as described in the manuscript.
Replication	Reproducibility of experimental findings were confirmed with technical and biological replicates of all experiments with the exception of 14C whole cell labeling (Supplementary), which was performed once due to substrate availability and time limitation.
Randomization	Randomization was not applicable to this study. Defined drug/substrate concentrations were required and all samples were analyzed.
Blinding	Blinding was not relevant to this study. Data was collected without bias - drug/substrate concentrations required in each experiment were not defined at data collection. Subsequently, defined drug/substrate concentrations were required upon data analysis.

Reporting for specific materials, systems and methods

We require information from authors about some types of materials, experimental systems and methods used in many studies. Here, indicate whether each material, system or method listed is relevant to your study. If you are not sure if a list item applies to your research, read the appropriate section before selecting a response.

Materials & experimental systems

n/a	Involved in the study	
[x]	[ ]	Antibodies
[x]	[ ]	Eukaryotic cell lines
[x]	[ ]	Palaeontology and archaeology
[x]	[ ]	Animals and other organisms
[x]	[ ]	Clinical data
[x]	[ ]	Dual use research of concern

Methods

n/a	Involved in the study	
[x]	[ ]	ChIP-seq
[x]	[ ]	Flow cytometry
[x]	[ ]	MRI-based neuroimaging
